# The complete chloroplast genome sequence of *Populus wilsonii* and its phylogenetic analysis

**DOI:** 10.1080/23802359.2017.1413291

**Published:** 2017-12-08

**Authors:** Xue-Min Han, Yi-Ming Wang, Yan-Jing Liu

**Affiliations:** a State Key Laboratory of Systematic and Evolutionary Botany, Institute of Botany, Chinese Academy of Sciences, Beijing, China;; b University of Chinese Academy of Sciences, Beijing, China;; c College of Biological Sciences and Biotechnology, Beijing Forestry University, Beijing, China

**Keywords:** *Populus wilsonii*, chloroplast genome, phylogenetic analysis, whole-genome sequencing

## Abstract

The complete chloroplast genome of *Populus wilsonii* was reconstructed by reference-based assembly using whole-genome sequencing data. The total chloroplast genome size of *P. wilsonii* was 158,080 bp in length, including a pair of inverted repeat regions (IRs) of 27,749 bp each, a large single-copy region (LSC) of 85,949 bp and a small single-copy region (SSC) of 16,633 bp. A total of 133 genes were predicted from the chloroplast genome, including 86 protein-coding genes, 39 tRNA genes and eight rRNA genes. Among these genes, 20 genes occurred in IRs, containing nine protein-coding genes, seven tRNA genes and four rRNA genes. The GC content of *P. wilsonii* chloroplast genome was 36.6%. The phylogenetic analysis with 15 other species showed that *P. wilsonii* was closely clustered with *Populus cathayana*. The complete chloroplast genome of *P. wilsonii* provides new insights into *Populus* evolutionary and genomic studies.

*Populus* is a genus of deciduous flowering plants, which was traditionally divided into six sections based on leaf and flower characters. *Populus wilsonii* is grouped into *Populus* section *Leucoides*, and its leaves are 8–20 cm long, 7–15 cm wide. Being native to China, *P. wilsonii* is found in Gansu, Hubei, Shaanxi, Sichuan, Xizang and Yunnan provinces. This poplar is related to *Populus lasiocarpa,* whose chloroplast genome was reported recently (Zhang et al. 2017). Given to both natural and artificial inter-specific hybrids, the classification of polar is very difficult. The genetic relationships and evolutionary history of poplar are still poorly investigated (Liu et al. 2016). The chloroplast genome was widely used in genetic and evolutionary studies (Rogalski et al. 2015). In this study, we reported the complete chloroplast genome sequence of *P. wilsonii*.

The sample of *P. wilsonii* was collected from Shennongjia Protected Area (Hubei, China; 31°41′40.38″N, 110°26′1.26″E). The total DNA was extracted from fresh leaves using the DNeasy Plant Mini Kit (Qiagen Inc., Valencia, CA) and then used for whole-genome sequencing on Hiseq 2500 platform (Illumina Inc., San Diego, CA). A total of 97.35 GB 100 bp paired-end raw reads were retrieved and then 67.81 GB quality-trimmed clean reads were used for the chloroplast genome reconstruction by the program MITObim v1.9 with default parameters (Hahn et al. 2013). The chloroplast sequence of *Populus trichocarpa* (NC_009143.1) was used as a reference (Tuskan et al. 2006). The chloroplast genome of *P. wilsonii* was annotated through the program DOGMA to predict protein-coding genes, transfer RNA (tRNA) genes and ribosome RNA (rRNA) genes (Wyman et al. 2004).

The chloroplast genome of *P. wilsonii* (GenBank accession no. MG214781) has a total length of 158,080 bp and comprises a small single-copy (SSC) region of 16,633 bp, a large single-copy (LSC) region of 85,949 bp, and a pair of inverted repeat (IR) regions of 27,749 bp each. It encodes 133 genes, including 86 protein-coding genes, eight ribosomal RNA genes and 39 transfer RNA genes. Most of these genes occurred in single copy in the SSC and LSC regions, while all genes were duplicated in the IR regions, including four rRNA species (*4.5S*, *5S*, *16S*, and *23S rRNA*), seven tRNA species (*tRNA-I^(CAU)^*, *-L^(CAA)^*, *-V^(GAC)^, -I^(GAU)^*,*-A^(UGC)^*, *-R^(ACG)^* and *-N^(GUU)^*) and nine PCG species (*rps19*, *rpl2*, *rpl23*, *ycf2*, *ycf15*, *ndhB*, *rps7*, *rps12, ycf1*). The overall GC content of the chloroplast genome was 36.6%, and the IR regions (41.9%) hold much higher GC content than the LSC (34.4%) and SSC (30.6%) regions.

To dip into the phylogenetic position of *P. wilsonii,* 16 complete chloroplast genomes were downloaded from NCBI, including 13 poplar and three other species. A maximum parsimony (MP) tree was constructed using MEGA 5.05 (Tamura et al. 2011). The phylogenetic tree showed that *P. wilsonii* and *Populus cathayana* were grouped together with a 100% bootstrap support (Figure 1). The complete chloroplast genome of *P. wilsonii* filled an important gap in providing chloroplast genome information for section *Leucoides*, providing further insights into *Populus* evolutionary and genomic studies.

**Figure 1.**
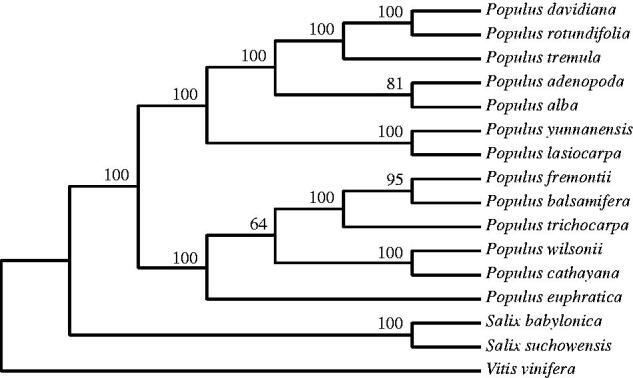
Phylogenetic tree of *Populus wilsonii* and other related species based on complete chloroplast genome sequences. The complete chloroplast genome sequences were downloaded from NCBI database and the phylogenetic tree was constructed by MEGA 5.05 software. The accession numbers of chloroplast genome sequence for this tree construction are listed as follows: *Populus davidiana* (NC_032717.1), *Populus rotundifolia* (NC_033876.1), *Populus tremula* (NC_027425.1), *Populus adenopoda* (NC_032368.1)*, Populus alba* (NC_008235.1), *Populus yunnanensis* (KP729176.1)*, Populus lasiocarpa* (KX641589), *Populus fremontii* (NC_024734.1), *Populus balsamifera* (NC_024735.1), *Populus trichocarpa* (NC_009143.1), *Populus cathayana* (KP729175.1), *Populus euphratica* (NC_024747.1), *Salix suchowensis* (NC_026462.1), *Salix babylonica* (KT449800), and *Vitis vinifera* (NC_007957.1).
